# Congressman Sheppard’s Defense of Old Men

**Published:** 1912-05

**Authors:** 


### Abstracts and Selections.

### Congressman Sheppard's Defense of Old Men

* * * It occurred to me that in the noisy onsweep of an intensely material era we were perhaps not sufficiently familiar with the capabilities of age. Indeed, sir, it has become too much a habit in recent years to disregard and put aside our older men and women. The clamor against old age, not only in the departments of the government, but in nearly all the other activities of the world, is absolutely senseless and unjust. This fact I hope to demonstrate in the short time now allotted to me. The idea has become too prevalent that after a certain age, by no means advanced, a man's usefulness diminishes as his years increase. A celebrated physician—Dr. Osler—expressed the opinion only a short while ago that the effective work of the world is done between the ages of 25 and 40. A more colossal error could not possibly have been made.

The impression has become entirely too general that our older men and women obstruct rather than facilitate the march of civilization. The truth is that the world owes infinitely more to men above the age of 50, an age ten years beyond the Osler limit, than to the men below it. Some two years ago an analysis was made by a scholar of accepted standing, Mr. Newman Dorland, of the lives and achievements of 400 foremost characters of human history. This analysis, to which I am indebted for many of the names I am about to present, showed that nearly 80 per cent of the world's greatest figures closed active lives between the ages of 50 and 80, 35 per cent continuing beyond 70, 22½ per cent beyond 80, 6 per cent beyond 90. Let us consider what has been achieved by men beyond the age of 80. Titian, master of Venetian painting, whose magic colors reflected the freshness and enthusiasm of a world saluting the return of art and learning, produced many of his most wonderful canvases after 80, painting his famous Battle of Lepanto at the age of 98. Fontenelle, one of the most versatile of men; Cornaro, the great disciple of temperance; Pope Leo XIII, John Adams, Theophrastus strode into the nineties with intellectual vigor unimpaired, Michael Angelo at 89 still held the sky a prisoner in his brush, having executed his Last Judgment, perhaps the most famous single picture in the world, and his celebrated frescoes in the Sistine Chapel between 60 and 70. See Von Moltke in full uniform at 88, still the chief of staff of the Prussian army; having crushed France at 72.

Hear John Wesley preaching with undiminished eloquence and power almost every day at 88, still directing the great religious movement he had founded, and closing amid unceasing activity at that remarkable age one of the most remarkable careers of his time, having traveled 250,000 miles in an age that knew neither electricity nor steam, delivered 4000 sermons, composed hundreds of volumes covering almost every phase of literature, earning through his publications $150,000, every cent of which he gave to charity during his life. * * *

See Guizot and Hobbes and Landor with active pens at 87. See Talleyrand and Thomas Jefferson, Herbert Spencer, Newton, and Voltaire, all fruitful in the eighties. See Bancroft, Buffon, and Ranke, writing deathless history after 80. See Palmerston, prime minister of England at 81, and John Quincy Adams, stricken in the fullness of his strength on the floor of Congress at the same age. Tennyson's "Crossing the Bar," the tenderest death song in our language, was composed at 83, Goethe's Faust at 80. See Gladstone conducting one of his most exciting political campaigns at 80, taking control of a nation and becoming its premier at 83. See Cato learning Greek; Plutarch, Latin; and Socrates, music, all at 80, and tell me no more that the old are no longer capable of high and useful achievement.

But let us proceed. Think of Joseph Jefferson portraying Rip Van Winkle with added effectiveness at 75, or the Irish actor, Macklin, actually taking part in a performance in England at 99. Think of Browning, brilliant and complex as ever at 77, or Whittier and Bryant issuing new volumes at 79. Think of Grimm, Laplace, Lamarck, completing tremendous tasks in the neighborhood of 80. Think of Perugino, at 76, painting the walls of a vast cathedral, or Humboldt deliberately postponing until 76 the best work of his life, his immortal Kosmos, completing it at 90. Think of Galileo discovering the daily and monthly vibrations of the moon at 73. Think of Irving and Lamartine, Hugo and Holmes, Wadsworth and Longfellow, Hallam and Grote, George Buchanan and Samuel Johnson, Kant, Savigny, and Littre, all astounding mankind with masterful productions between 70 and 80. Think of Henry Clay, Calhoun, Metternich, Bismarck, Crispi, Thiers, Franklin, Morgan, Reagan, Roberts, Allison Morrill, Cannon, all towering figures in politics after 70. Think of Commodore Vanderbilt increasing the mileage of his railroads from 120 to 10,000, adding a hundred millions to his fortune, between 70 and 88.

Turning to the period from 60 to 70, the list grows still more interesting and comprehensive. To this decade belong the best deductions of Confucius; Bismarck's inauguration of a colonial career for Germany; Pasteur's discovery of a cure for hydrophobia; Monroe's famous doctrine for the protection of the South American republics, the permanent safeguard of a continent's liberties; the third and fourth voyages of Columbus, resulting in the discovery of South America; and many of the brightest deeds of Webster, Beaconfield, Jefferson, John Adams, Benjamin Franklin, and Martin Luther. To this period belong many of the world's most splendid paintings. In music some of the rarest fabrics of Wagner, Haydn, Verdi, and Gounod are the fruitage of this period. In general literature, philosophy, and science many of the most imposing performances have been achieved by authors between 60 and 70. Prominent among these we find many of the best compositions of Cervantes, Schopenhauer, Hugo, John Stuart Mill, Berkley, Mommsen, Voltaire, Ruskin, Emerson, and Francis Bacon. Especial mention should be made of Michelet's great history of France, Dryden's ode on St. Cecelia's day, Milton's Paradise Regained and Samson Agonistes, Edmund Burke's Reflections on the Revolution in France, and Sir Richard Burton's translation of Arabian Nights, a source of infinite delight to every English-speaking fireside.

Coming now to the deeds of men between 50 and 60 we find many of the most far-reaching achievements of all history. Between 50 and 60 Columbus made his first voyages of American discovery, perhaps the most important single event in human records; Marlborough won Blenheim, Morse invented the telegraph, Richelieu reconstructed France, Cæsar corrected the calendar and wrote his Commentaries, Cromwell established the protectorate, Lincoln issued the emancipation proclamation, Bright instituted his reforms, Loyalo founded his great society, Jefferson the democracy, Knox accomplished a great religious revolution. Wyclif and Luther translated the Bible and brought its eternal truths to the hearts and hearths of the English and German masses, Schliemann made his most notable excavations, Hunter gave a fundamental impetus to surgery, Kepler contrived his table of logarithms, Chesterfield his system of social ethics, Hegel and Lotze their systems of philosophy, Leibnitz founded the Academy of Berlin, Penn negotiated his famous treaty with the Indians, Washington became the first President of the United States, Robert E. Lee made the Confederate resistance sublime, Herschel invented the reflecting telescope, Canning and Peel performed their most brilliant labors, Burke devised his India bill and secured the impeachment of Warren Hastings, Garibaldi became the ruler of Italy.

Between 50 and 60 Leidy made his most valuable contributions to biology, Cuvier to natural history; Copernicus wrote his great treatise on the revolution of celestial bodies, Adam Smith his "Wealth of Nations," the foundation of modern political science. Between 50 and 60 Plato and Aristotle gave their principal creations of the world. Between 50 and 60 Kant wrote the "Critique of Pure Reason," Bacon the "Novanum Organum," and Locke the "Essay on the Human Understanding," each of these three great works being veritable pillars of modern learning and progress. Between 50 and 60 were written Bunyan's "Holy War" and the second part of "Pilgrim's Progress," Boswell's "Life of Johnson," De Foe's "Robinson Crusoe," Dante's "Divine Comedy," Milton's "Paradise Lost," Chaucer's "Canterbury Tales," the first part of Cervante's "Don Quixote," the second part being written after 60; "La Fontaine's Fables," "Gulliver's Travels," all treasures that will enrich the literature of the world forever. The average age of the Chief Justices of the Supreme Court of the United States, perhaps the greatest legal tribunal on earth, is nearer 70 than 60, Marshall having concluded his prodigious labors of more three three decades at 80, Taney at 88, Waite at 72, Fuller still presiding over that august body today at 76. It is safe to say that the average age at which all the more than fifty Associate Justices who have occupied the Supreme Bench since its organization were still in the full exercise of their functions at nearer 65 than 60.

Such is a partial list of the achievements of men who have passed the half-century mark. Eliminate these achievements and you would blot out most of the world's advancement. Observe that we are still ten years above the Osler limit of 40. Were we to go still further, and erase the deeds of the "infants" between 40 and 50, we would destroy about all that remains of human progress. We should have to eliminate the printing press of Gutenberg, the discoveries in electricity of Franklin and Galvani, Priestley's discovery of oxygen, the smallpox preventives of Jenner, Marvey's discovery of the circulation of the blood, La Salle's discovery of the Mississippi, Bessemer's process for the manufacture of steel. Watt's steam engine, Stephenson's railways, the military feats of Grant and Sherman and Cromwell and Nelson, Blackstone's Commentaries on the Principles of English Law, the services of Washington in the American Revolution. In science, music, art, and literature we would wipe out almost all the surviving contributions to human enlightenment; among these the conceptions of Herschel and Von Baer, the creations of Liszt and Sphor, the creations of Dore and Aubens and Blake, many of the masterpieces of Shakespeare, Scott, Gibbon, Hume, Macaulay, Carlyle, Petrarch, Pope, Dickens, Chateaubriand, Lessing, Spurgeon, Dumas, Milman, Motley and Gray. This brief and incomplete review will show that our older men are by no means to be despised; that we owe to them what is most permanent and uplifting in the civilization of the world. It shows that a man rarely reaches the full fruition of his powers until he enters the forties and the fifties, and that as long as life throbs within his bosom he is capable of tremendous service to mankind, that "age is opportunity no less than youth itself."

In the words of Mr. James Q. Howard, one of the most gifted officials in our congressional library, himself an example of the possibilities of age, a man is as a rule "immature, unripe, callow, vealy, verdant, sappy, bumptious, bat-blind, and grass-green until he reaches the age of 40 years." I repeat that there has been of late too much of a disposition to neglect and disregard the old. I would not deprecate the encouragement of young men and women, they have a high and effective mission to fulfill; but the old need encouragement as well; affection and solicitude are as welcome to their twilight years as to the radiant hours of the young. Beneath gray locks may blaze the fires of genius; perhaps a gentle word may wake in feeble eyes the vision of an eagle. Society in its fatuous adulteration of mere youth is falling into serious error. Such an attitude is a contradiction of the truth of history, a violation of all the teachings of experience. There are advantages of which age alone may boast. The passions that lashed the early years now lie obedient at the feet of reason. The impulses that stirred the youthful soul to violence and sin are sleeping in the cradle of a mature philosophy. No more does anger break in curses on the lips or envy hiss its shameful whispers. Revenge no longer prompts the uplifted arm; on the venerable countenance there broods prophetic peace.

The weaknesses of men and governments stands out in startling contrast with the ideals experience alone develops and age alone may understand. Contemplation imparts a glory to the furrowed brow as in the silent sunset of a noble life the storms and follies of the world become mere distant echoes. Society must learn again that services of unmeasured value may be rendered by the old. It must learn again that in neglecting the old it is wasting one of its most valuable assets. It is the general complaint of students of human institutions that each generation repeats in large measure the blunders of former ones; that if each generation could begin at the exact point in knowledge and experience where the other left off the progress of the world would be wonderfully accelerated. This complaint would have far smaller basis if we but learned to heed and love and glorify our older men and women.

And, after all, old age is but a fiction; there is no old age of the soul.

> The hands of youth are smooth and beautiful
> And round and finely formed and white and cool.
>
> But I have known two old and twisted hands
> With knotted veins and fingers bent with work;
> No grace of form is left those wasted frames
> Wherein the hidden grace of life doth lurk.
>
> But thin and old and cramped, they on them bear
> The marks and signs of those who suffer much;
> The patient strength of all the earth is theirs,
> And tenderness untold is in their touch.
>
> The hands of youth are smooth and white with ease,
> But God hath clasped such twisted hands as these.

O, sir, lét the crusade against our older men and women cease. The world needs and Heaven consecrates the ripened wisdom of the mellow year.

